# Level of invasion into fibromuscular band is an independent factor for positive surgical margin and biochemical recurrence in men with organ confined prostate cancer

**DOI:** 10.1186/s12894-018-0321-z

**Published:** 2018-02-02

**Authors:** Aram Kim, Myong Kim, Se Un Jeong, Cheryn Song, Yong Mee Cho, Jae Yoon Ro, Hanjong Ahn

**Affiliations:** 1 Department of Urology, Asan Medical Center, University of Ulsan College of Medicine, 88 Olympic-Ro 43 Gil Songpa-Gu, Seoul, 05505 Republic of Korea; 2 Department of Pathology, Asan Medical Center, University of Ulsan College of Medicine, Seoul, 05505 Republic of Korea; 3 Department of Pathology and Genomic Medicine, Houston Methodist Hospital, Weill Medical College of Cornell University, Houston, TX 10065 USA; 4 Department of Urology, Konkuk University Medical Center, Konkuk University School of Medicine, Seoul, 05030 Republic of Korea

**Keywords:** Organ confined prostate cancer, Biochemical recurrence, Positive surgical margin, Fibromuscular band, Predictive factor

## Abstract

**Background:**

This study aimed investigate the effect of the level of invasion into fibromuscular band (FMB) of prostate on the positive surgical margin (PSM) and biochemical recurrence (BCR) after radical prostatectomy (RP) in patients with organ-confined (pT2) prostate cancer.

**Methods:**

The clinical and pathological data of 461 consecutive patients with pT2 prostate cancer were evaluated regarding the level of invasion into FMB. The relationship between levels of invasion into FMB and PSM / BCR was assessed.

**Results:**

The rate of PSM at an FMB level of at 2 was 18.8%, which was significantly greater than the rates at levels 0 (5.4%) and 1 (7.8%). The level of FMB (*p* = 0.007) and percentage of tumor volume (*p* = 0.012) were identified as independent factors predictive of a positive surgical margin in a multivariate analysis. The 5-year BCR-free survival rates for a level 0–1 FMB with negative surgical margin, level 0–1 FMB with positive surgical margin, level 2 FMB with negative surgical margin, and level 2 FMB with positive surgical margin were 96.6%, 86.4%, 85.6%, and 72.9%, respectively (*p* <  0.001). A level 2 FMB (*p* = 0.050), positive surgical margin (*p* = 0.001), and surgical Gleason score (*p* = 0.001) were identified as independent predictors of a BCR of pT2 prostate cancer.

**Conclusions:**

Among patients with negative surgical margins, the surgical Gleason score and level of FMB independently affected the incidence of a BCR of pT2 prostate cancer. The level of FMB was an independent predictor of both a positive surgical margin and a BCR of pT2 disease. Accordingly, the level of FMB might help to further stratify the prognosis of patients with pT2 disease.

## Background

All oncologic surgeries aim to completely remove cancers. Therefore, the presence of a positive surgical margin (PSM) after radical prostatectomy (RP) for prostate cancer is considered an adverse event in curing this cancer, with outcome associated with prostate specific antigen (PSA) biochemical recurrence (BCR) and poor outcome [1, 2]. The reported rates of PSMs among pathologically localized prostatectomy specimens vary from 6.5% to 38% in contemporary series of RP [1, 3]. The rate of PSM for pT2 prostate cancers is thought to reflect the surgeon’s experience [4, 5]. However, tumor-behavioral factors might also influence the rate of PSM for organ-confined prostate cancer. A large-volume tumor adjacent to the prostatic capsule may be prone to a PSM if the surgeon rigorously attempts to preserve the nerves and maximize the remaining functioning urethra. The level of prostatic capsular invasion, which focuses on the extra-prostatic extension (EPE), was reported to affect the incidence of BCR [6]. However, recent articles have avoided the term “prostatic capsule” [6–8], as the prostate does not have a true capsule at the apex, anterior side, and base. Therefore, we instead revisited the level of invasion into fibromuscular band (FMB) as an independent factor for a PSM or BCR in patients with organ-confined prostate cancer. We hypothesized that the level of invasion into FMB would be an independent tumor-behavioral factor that could affect the rates of PSM and BCR in patients with pT2 prostate cancer.

## Methods

### Patient selection

We reviewed 473 patients with pT2 prostate cancer who had undergone RP (robotic: 369, open: 104) at our hospital from January 2010 to March 2014. We excluded four patients who received neoadjuvant treatment and eight patients for whom complete clinical data were unavailable or who were lost to follow-up. Eventually, 461 patients were enrolled, with a median follow-up of 51.2 (range: 1.9–74.9) months. The serum PSA levels were measured at 3-month intervals during the first year after RP, 6-month intervals during years 2–5, and annually thereafter. A BCR was defined as a PSA level > 0.2 ng/mL.

### Pathologic evaluation and determination of the level of invasion into fibromuscular band

**Fig. 1:**
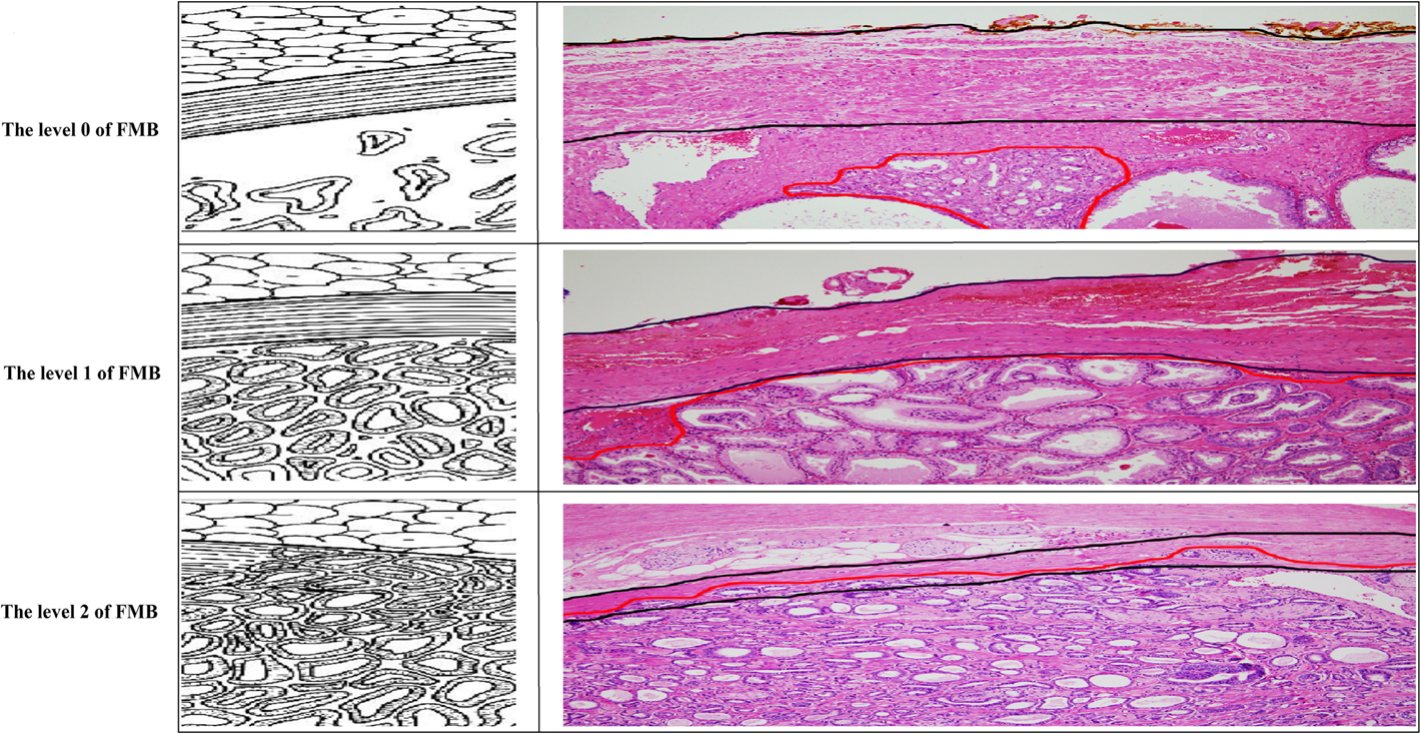
Illustration and microscopic images of the level of invasion into fibromuscular band (black line: fibromuscular band; red line: prostate cancer)

All surgical specimens were assessed microscopically after histological sectioning. Each specimen was weighed, and the external surface was covered in India ink prior to fixation in 10% formalin. Each specimen was examined in 3–5 mm sections from the base to the apex, perpendicular to the major. Subsequently, the sections were divided into halves or quadrants to fit the cassettes routinely used for paraffin embedding, and slide-mounted thin sections were stained with hematoxylin-eosin. Primary and secondary Gleason scores were assigned to the total cancer within the specimen, according to the revised (2005) criteria for Gleason scoring [9]. Each specimen was staged according to the 2010 American Joint Committee on Cancer staging system [10]. The presence of cancer cells in the inked surface of RP specimen was considered a positive surgical margin [11]. The percentage of tumor volume (PTV) was estimated by the sum of visually determined tumor foci relative to the prostate gland on every section, and categorized into three groups: < 5%, 5–15.0%, and > 15.0%. The levels of EPE were classified as 0, 1, and 2 according to the classification by Wheeler et al. (Fig. 1) [12]. In level 0, the cancer cells are located in the prostatic stroma with a normal gland. Level 1 involves cancer cells within the prostatic stroma but beyond the boundary of normal gland. Level 2 involves cancer cells confined to the prostate, within a layer more fibrous than muscular. We used the highest level of FMB if there were several tumors in prostate and the levels of FMB were evaluated and confirmed by 2 pathologists.

### Statistical analysis

The patients’ clinical and pathological characteristics were compared according to the levels of EPE, using the *χ*^2^ test (categorical variables) and Student’s *t*-test (continuous variables). Uni- and multivariate binary logistic regression analyses were performed to determine the pre- and post-operative variables predictive of a PSM. The 5-year BCR-free survival rate after RP was estimated using the Kaplan–Meier method. Cox proportional hazard analyses were performed to identify the prognostic factors affecting the incidence of BCR. All statistical analyses were performed using commercially available software (SPSS® version 21.0, IBM, Chicago, IL, USA), and a *p* value < 0.05 was considered statistically significant.

## Results

### Comparison of clinical features according to the level of invasion into fibromuscular band

The patients’ clinical and pathological characteristics are summarized in Table 1. A higher clinical T stage, percentage of positive cores, and biopsy Gleason score were more frequently observed among patients with level 2 FMB, compared to those with levels 0 and 1. The PTV increased significantly according to the level of FMB, and higher surgical Gleason scores were more frequent with a level 2 FMB than with a level 0 or 1 FMB (all *p* <  0.001). Other parameters such as the PSA and prostate size did not differ significantly among the three groups (p range, 0.426–0.672; Table 1).

**Table 1:** Clinical and pathological characteristics of the patients with pT2 prostate cancer

Level of FMB	Total	Level 0	Level 1	Level 2	*p* value
No. of patients	461	74	243	144	
Mean PSA (ng/mL)	6.6	6.5	6.5	7.0	0.672
Prostate size (cc)	36.0	37.8	35.2	36.5	0.426
Mean % of positive biopsy cores	20.2	13.0	19.0	26.2	< 0.001
Clinical stage					0.031
cT1	257	53	130	73	
≥ cT2	204	21	113	71	
Biopsy Gleason score, No.					< 0.001
6	197	50	104	43	
3 + 4	160	19	84	57	
4 + 3	71	2	38	31	
8–10	33	3	17	13	
Surgical Gleason score, No.					< 0.001
6	124	41	65	18	
3 + 4	205	26	110	69	
4 + 3	99	4	51	44	
8–10	33	3	17	13	
Mean PTV	8.7	3.5	8.4	11.5	< 0.001
Rate of PSM, N (%)	50 (10.8%)	4 (5.4%)	19 (7.8%)	27 (18.7%)	0.001

*FMB* fibromuscular band, *PSA* prostate specific antigen, *PTV* percentage of tumor volume, *PSM* positive surgical margin

### Associations between the level of invasion into fibromuscular band and PSM after RP

The rates of PSM at a level 2 FMB (18.7%) were significantly greater than those at levels 0 (5.4%) and 1 (7.8%) (*p* = 0.001), whereas this parameter did not differ significantly between levels 0 and 1 (*p* = 0.495; Table 1). In a univariate analysis with postoperative variables, the PTV (p = 0.001) and level of FMB (*p* = 0.001) were significant factors affecting the PSM. The PTV (*p* = 0.012) and level of EPE (*p* = 0.007) were also identified as independent factors in a multivariate analysis (Table 2).

**Table 2:** Risk factors for PSM after radical prostatectomy in pT2 prostate cancer

	Univariate analysis		Multivariate analysis	
	OR (95% CI)	*p* value	OR (95% CI)	*p* value
PSA	1.036 (0.994–1.080)	0.092		
Level of FMB		0.001		0.007
Level 0–1	Reference		Reference	
Level 2	2.817 (1.561–5.083)		2.303 (1.252–4.235)	
PTV		0.001		0.012
Lesser than 5%	Reference		Reference	
5–15.0%	1.793 (0.814–3.948)	0.147	1.600 (0.720–3.557)	0.249
Greater than 15.0%	4.147 (1.863–9.231)	< 0.001	3.293 (1.447–7.498)	0.005
Surgical Gleason score		0.130		
6	Reference			
3 + 4	2.178 (0.909–5.219)	0.081		
4 + 3	3.092 (1.218–7.849)	0.018		
8–10	2.266 (0.621–8.267)	0.215		

*PSA* prostate specific antigen, *FMB* fibromuscular band, *PTV* percentage of tumor volume, *PSM* positive surgical margin

### Associations between the level of invasion into fibromuscular band and BCR-free survival after RP

**Fig. 2:**
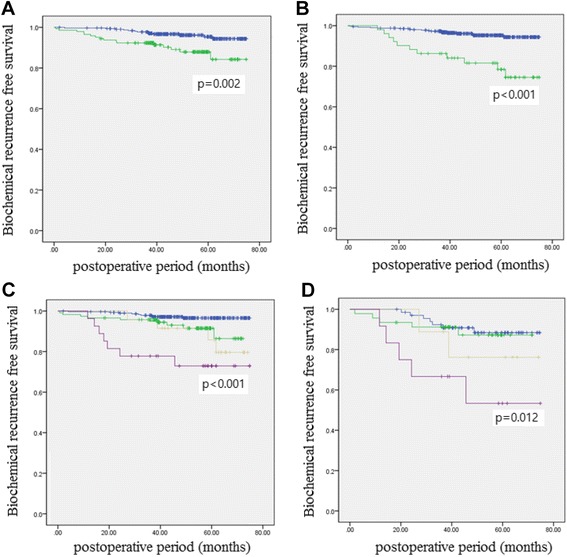
Biochemical recurrence-free survival over time in patients with pT2 prostate cancer and (**a**) a level 0–1 FMB (Blue) vs. level 2 FMB (Green); **b** negative surgical margin (Blue) vs. positive surgical margin (Green); **c** level 0–1 FMB and NSM (Blue) vs. level 0–1 FMB and PSM (Green) vs. level 2 FMB and NSM (Yellow) vs. level 2 FMB and PSM (Purple); **d** Among patients with a surgical Gleason score > 4 + 3, a level 0–1 FMB and NSM (Blue) vs. level 0–1 FMB and PSM (Green) vs. level 2 FMB and NSM (Yellow) vs. level 2 FMB and PSM (Purple)

During the follow-up period, 29 patients (6.2%) experienced a BCR at a mean interval of 28.8 months after RP. Overall, the 5-year BCR-free survival rate was 92.4%. The 5-year BCR-free survival rates of patients with level 0–1 FMB and level 2 FMB were 95.4% and 84.2%, respectively (*p* = 0.002; Fig. 2a), and those of patients with a negative surgical margin and PSM were 94.4% and 78.5%, respectively (*p* <  0.001; Fig. 2b). The 5-year BCR-free survival rates of patients with Gleason scores of 6, 3 + 4, 4 + 3 and 8–10 were 97.6%, 94.5%, 86.7%, and 76.6%, respectively (p <  0.001).

When patients were stratified by margin status and level of FMB (level 0–1 vs. level 2), the 5-year BCR-free survival rates of those with a level 0–1 FMB and negative surgical margin, level 0–1 FMB and PSM, level 2 FMB and negative surgical margin, and level 2 FMB and PSM were 96.6%, 86.4%, 85.6%, and 72.9%, respectively (*p* < 0.001; Fig. 2c). Patients with a level 0–1 FMB and PSM did not significantly differ from those with a level 2 FMB and negative surgical margin in terms of survival (*p* = 0.578). Among the subgroups with surgical Gleason scores greater than 4 + 3, the corresponding survival rates were 88.4%, 87.2%, 76.2%, and 53.3%, respectively (*p* = 0.012; Fig. 2d).

A multivariate analysis of postoperative variables predictive of a BCR identified the surgical Gleason score (*p* = 0.001), PSM (p = 0.001), and level 2 FMB (*p* = 0.050) as independent predictors (Table 3). Among the subgroups with negative surgical margins, the level of FMB remained an independent prognostic factor for a BCR (*p* = 0.045), along with the surgical Gleason score (p = 0.001) and PSA level (*p* < 0.001).

**Table 3:** Risk factors for BCR after radical prostatectomy (RP) in pT2 prostate cancer

	Univariate analysis		Multivariate analysis	
	HR (95% CI)	*p* value	HR (95% CI)	*p* value
Level of FMB		0.003		0.050
Level 0–1	Reference		Reference	
Level 2	3.067 (1.471–6.393)		2.124 (1.001–4.508)	
Surgical Gleason score		< 0.001		0.001
6	Reference		Reference	
3 + 4	2.417 (0.1513–11.383)	0.264	1.878 (0.395–8.923)	0.428
4 + 3	7.909 (1.770–35.348)	0.007	5.585 (1.229–25.384)	0.026
8–10	14.099 (2.929–67.876)	0.001	10.311 (2.119–50.175)	0.004
PTV		0.084		
Lesser than 5%	Reference			
5–15.0%	2.421 (0.872–6.723)	0.090		
Greater than 15.0%	3.364 (1.150–9.841)	0.027		
Surgical Margin Status		< 0.001		0.001
Negative	Reference		Reference	
Positive	4.813 (2.268–10.213)		3.607 (1.683–7.726)	

*BCR* biochemical recurrence, *FMB* fibromuscular band, *PTV* percentage of tumor volume

## Discussion

Although the effect of a PSM on long-term cancer-related mortality remains controversial [2, 13], this factor has been proposed as an important predictor of disease progression [14, 15] and one of the strongest determinants of the possibility of benefitting from adjuvant radiotherapy [1]. Among pT2 prostate cancers, a PSM has been considered an iatrogenic factor because these tumors are confined to the prostate; therefore, the surgeons’ experience might be important for reducing the incidence of PSM in a prostate-confined cancer [4, 5]. Previous studies have reported dramatically different rates of PSM according to the surgeons’ clinical experience [16], and found that the learning curves for surgical margins after open or laparoscopic RP plateaued at approximately 200–250 cases [17, 18]. We also reported a similar experience regarding the learning curves for surgical margins in a robotic RP series, wherein we demonstrated that the robotic RP reached a comparable PSM rate for pT3 disease with a surgical experience exceeding 500 cases [19]. However, even after transcending this learning curve for surgical margins, surgeons are among organ-confined prostate disease. Tumor-behavioral factors, such as the extent of EPE, have been identified as risk factors for a PSM, along with the preoperative PSA level and Gleason score [4, 20]. However, it remains unclear whether the tumor-behavioral factor itself is important in terms of the PSM. We previously identified the PTV as an independent risk factor for a PSM (*p* = 0.035), and both the PTV (*p* < 0.001) and surgical Gleason score (≥8; *p* = 0.021) as independent predictors of a BCR of organ-confined prostate cancer [21].

In this study, we hypothesized that the level of invasion into FMB might be another important tumor-behavioral factor associated with the risks of PSM and BCR after a RP of organ-confined disease. Our current results demonstrated that the level of invasion into FMB was an independent risk factor for a PSM (level 2 PCI; *p* = 0.007), along with the tumor volume (*p* = 0.013; Table 2). Accordingly, the level of invasion into FMB, tumor volume, and surgical Gleason score are tumor-behavioral factors that might promote a PSM after RP for organ-confined prostate cancer. On the whole, these results agree with those in our previous reports [21]. However, the significance of PTV for BCR was not consistent with our previous study, which included an older cohort (2000–2007) and used specialized method of PTV estimation [21]. The mean PTV was higher in our previous study than in the present study (12.1% vs. 8.7%).

We believe that the level of invasion into FMB has important clinical implications for organ-confined prostate cancer. Although a previous study reported that higher levels of prostatic capsular invasion were associated with a more adverse prognosis among patients with prostate cancer, the authors did not demonstrate whether the level of prostatic capsular invasion was an independent prognostic factor for a BCR of pT2 prostate cancer [6]. In our study, we used the term “fibromuscular band (FMB)” which was commonly accepted instead of “prostatic capsule” [6] and identified the level of invasion into FMB as an independent predictor of a BCR (Table 3). The 5-year BCR-free survival rate among patients with a level 2 FMB and negative surgical margin was similar to that of patients with a level 0–1 FMB group and PSM (86.4% vs. 85.6%; *p* = 0.578; Fig. 2c). Especially, patients with pT2 disease, a level 2 FMB, PSM, and a surgical Gleason score ≥ 4 + 3 had a worse 5-year BCR-free survival rate, compared to patients with a level 0–1 EPE, PSM, and surgical Gleason score ≥ 4 + 3, although this difference failed to reach statistical significance (53.3% vs. 76.2%; *p* = 0.225). The recent American Urology Association (AUA)/American Society for Therapeutic Radiology and Oncology (ASTRO) guideline mandated the use of adjuvant radiation therapy for patients with pT3 prostate cancer, based on the results from several randomized controlled trials [22]. Moreover, several studies of non-organ confined prostate cancer demonstrated that adjuvant radiotherapy significantly reduced the risk of BCR and improved metastasis-free and overall survival [23–25]. Although RP provides decent overall long-term oncologic control and favorable survival rates in patients with organ-confined prostate cancer, we note that the clinical courses might vary depending on the presence of other risk factors. Given the BCR-free survival rates reported for the control arms of previous studies [23–25], we believe that a certain subgroup of patients with pT2 disease will require adjuvant radiotherapy to reduce the risk of BCR after RP. The group with the worst prognosis in the present study, those with pT2 disease, a level 2 FMB, PSM, and surgical Gleason score ≥ 4 + 3, had a 5-year BCR-free survival rate of 53.3%, similar to that of patients with T3b disease in a previous study [26]. We believe that these patients should therefore be considered candidates for adjuvant radiotherapy after RP.

The present study had a retrospective design and relatively small sample size, and there were other limitations, such as a critical reproducibility of the level of invasion into FMB. Pathologists must re-evaluate the level of invasion into FMB, as well as the levels of all tumors. The highest level of FMB should be used for cases involving multiple tumors in the prostate. Furthermore, the effect of the level of invasion into FMB according to the tumor location remains controversial, and additional studies are needed. Despite these limitations, our study attempted to revisit the concept of the level of invasion into FMB as a tumor-behavioral factor in a contemporary RP cohort. External validation studies with larger cohorts or prospectively designed studies are needed to confirm the clinical implications of our findings.

## Conclusions

The level of invasion into FMB is an independent tumor-behavioral factor for PSM and BCR in organ-confined prostate cancer. Patients with a level 2 FMB had a higher risk of BCR after RP. Therefore, the level of invasion into FMB might be used to stratify patients with pT2 disease and a poor prognosis.

## Abbreviations

BCR: Biochemical recurrence
EPE: Extra-prostatic extension
FMB: Fibromuscular band
PSA: Prostate specific antigen
PSM: Positive surgical margin
PTV: Percentage of tumor volume
RP: Radical prostatectomy
